# Perceptions and Knowledge of Autism Spectrum Disorder in Primary Care and Educational Settings: A Study From Central Portugal

**DOI:** 10.7759/cureus.86986

**Published:** 2025-06-29

**Authors:** Ana Rita Fradique, Sofia Bodas de Carvalho, Arminda Jorge

**Affiliations:** 1 Department of Pediatrics, Coimbra Local Health Unit, Coimbra Pediatric Hospital, Coimbra, PRT; 2 Faculty of Health Sciences, University of Beira Interior, Covilhã, PRT; 3 Department of Pediatrics, Cova da Beira Local Health Unit, Covilhã, PRT

**Keywords:** autism, family physicians, kindergarten educators, professionals’ knowledge, teacher

## Abstract

Introduction: Autism spectrum disorder (ASD) is a neurodevelopmental condition that typically manifests in early childhood and is ideally identified during the first years of life. Family physicians, primary school teachers, and kindergarten educators play a crucial role in the early detection of neurodevelopmental disorders, which is essential for improving outcomes. This study aimed to assess professionals’ knowledge and perceptions regarding ASD.

Methods: A cross-sectional, quantitative study was conducted with a convenience sample of family physicians, primary school teachers, and kindergarten educators in the Cova da Beira region in central Portugal. A questionnaire on ASD, adapted from a previously validated instrument and updated based on current literature, was used. Descriptive statistical analysis and two-way frequency tables were used to explore relationships between variables. The chi-square test was applied to assess differences between independent groups. A p-value of <0.05 was considered statistically significant.

Results: A total of 95 professionals responded to the questionnaire: 57 were primary school teachers, 21 were kindergarten educators, and 17 were family physicians; 80% of participants (n=76) reported not feeling confident in identifying early warning signs or monitoring children with ASD. Half of the respondents believed that symptoms typically appear before the age of three. Among family physicians, none fully agreed with being able to identify early signs. However, most respondents expressed interest in receiving training in this area.

Conclusion: There is a clear need to invest not only in the academic training of future professionals but also in continuing education strategies to address the existing knowledge gaps among currently practicing professionals in the field of autism.

## Introduction

Autism spectrum disorder (ASD) is a neurodevelopmental condition characterized by persistent deficits in social communication and social interaction across multiple contexts, accompanied by restricted and repetitive patterns of behavior, interests, or activities, as defined by the Diagnostic and Statistical Manual of Mental Disorders, Fifth Edition (DSM-5) [1]. The global prevalence of ASD has been increasing steadily [2,3], currently affecting an estimated one in 36 children in the United States, with no significant differences across ethnicity or socioeconomic status and a fourfold higher prevalence among males [4]. Prevalence rates vary by region, ranging from 0.4% in Asia to 1.7% in Australia, with intermediate values in Europe (0.5%) and the Americas and Africa (1%) [3]. In mainland Portugal, a 2007 study estimated a prevalence of 9.2 per 10,000 children aged between six and nine years, with the highest rates reported in the central region (12.5/10,000) [5]. The Cova da Beira region, located in central Portugal and characterized by its predominantly rural setting, may present specific challenges that influence both ASD diagnosis rates and the level of professional awareness.

The etiology of ASD is multifactorial, involving genetic predisposition and environmental factors such as prenatal stress, infections, nutritional deficiencies, and exposure to pollutants during pregnancy [2,6-8]. While the exact mechanisms remain unclear [8], recent findings by Rolland et al. [9] show that rare loss-of-function genetic variants are associated with lower cognitive performance and socioeconomic outcomes, even in undiagnosed individuals, reinforcing the role of genetics in ASD.

The concept of a "spectrum" reflects the wide variability in symptom presentation, including differences in the type, intensity, and severity of symptoms. Early warning signs of ASD can often be observed between 12 and 24 months of age [10,11], and in some cases, as early as the first 6 months of life [10,12]. Social communication and emotional expression are typically altered, with deficits such as poor eye contact, absence of declarative gestures, and lack of response to name or voice [2,10,13,14]. Additional signs include deficits in joint attention, a lack of symbolic play, and limited imitation skills [2,10]. While language delay is not exclusive to ASD, it is one of the main reasons parents seek medical evaluation (60% to 70% of children with ASD exhibit some form of language delay) [10]. Children with ASD may prefer solitary play and often exhibit greater interest in objects than in people [2,10]. Repetitive and stereotyped movements, whether self-directed or involving objects, are common, as are echolalia and strong attachment to routines, with marked resistance to change [2,10,15,16]. Motor deficits, including hypotonia, abnormal posture, and either hyperactivity or hypoactivity, are also frequently reported [9,13,15]. Sensory processing abnormalities are common, affecting auditory, visual, tactile, and oral modalities [17].

ASD is frequently associated with other comorbidities, such as intellectual developmental disorder (IDD) in 30% to 50% [12] and epilepsy (5% to 30%) [15], gastrointestinal issues (30% to 70%) [18], sleep disturbances (50% to 80%) [19], attention deficit hyperactivity disorder (ADHD) in 30% to 60% [20], and depression (10% to 30%) [15], anxiety (20-40%) [20], and self-injurious behaviors (15% to 20%) [19]. Despite these complex clinical presentations, ASD remains a clinical diagnosis, typically supported by standardized assessment tools such as the Autism Diagnostic Interview-Revised (ADI-R) and Autism Diagnostic Observation Schedule, Second Edition (ADOS-2) [21,22]. Several screening instruments are available [6], with the Modified Checklist for Autism in Toddlers, Revised (M-CHAT), being particularly notable for use in routine consultations [23]. Beyond parental observations, family physicians and early childhood educators are in a privileged position to detect developmental concerns early, allowing for the timely identification of warning signs and appropriate referral to specialized services [6,23].

Non-pharmacological interventions, particularly training in social and daily living skills, constitute the foundation of ASD management, with early detection significantly improving long-term outcomes [21,23]. Early intervention also enables caregivers to address their concerns and uncertainties while facilitating the development of appropriate support strategies for both the child and their environment [6,23]. Community awareness is equally important, as it promotes social inclusion and facilitates educational and workplace integration for individuals with ASD [6]. Pharmacological treatment may be considered for the management of associated comorbidities, particularly disruptive behaviors and sleep disturbances [2], in cases where non-pharmacological approaches are insufficient [6,18].

Although diagnostic and intervention protocols for ASD are available, early detection remains essential. Timely identification optimizes developmental outcomes. However, limited data are available regarding the knowledge and awareness of healthcare and education professionals about ASD. Therefore, the aim of this study was to assess the knowledge, perceptions, and difficulties reported by family physicians, primary school teachers, and kindergarten educators concerning ASD, specifically regarding early warning signs, diagnosis, etiology, prognosis, and the challenges associated with managing and supporting children with this condition.

## Materials and methods

This was a cross-sectional, quantitative, observational, and correlational study conducted between January and March 2016. The target population consisted of family physicians and family practice residents from the Cova da Beira Health Center Group (ACeS Cova da Beira), as well as kindergarten educators and primary school teachers from the educational groupings in Cova da Beira, central Portugal. The only exclusion criterion was refusal to participate.

Collaboration requests were initially sent via email and followed by phone calls to five Personalized Health Care Units (UCSPs) affiliated with ACeS Cova da Beira and 12 school groupings (including kindergartens).

The data collection instrument used in this study was a structured questionnaire designed to assess knowledge and attitudes about ASD. It was adapted from a tool originally developed by Christian Müller, a Brazilian neuropediatrician and researcher, in the context of a 2012 thesis conducted at the Federal University of Rio Grande do Sul [24]. Although the original questionnaire was not published as a standardized scale with a version number or formal licensing, it is freely available for academic use. The questionnaire was translated and culturally adapted to the Portuguese context, supported by a literature review, and pre-tested with three individuals (a family physician and two teachers) with similar professional and educational backgrounds to the target population. The pilot aimed to evaluate item clarity, cultural relevance, and applicability, leading to minor wording adjustments. The final version of the questionnaire included multiple-choice items organized into three thematic areas: demographic data (sex, age, profession), knowledge about ASD (including early signs, age of onset, diagnostic criteria, etiology, and prognosis), and professional experience with ASD (including contact frequency and prior training). An optional open-ended section titled “Observations/Suggestions” allowed participants to provide additional reflections beyond the structured format. The questionnaire was administered in paper format and delivered by hand at the participants’ workplaces (Appendices A, B). This in-person distribution strategy was chosen to facilitate access and maximize response rates among healthcare and education professionals. All participants completed the questionnaires anonymously and independently after receiving a brief explanation of the study’s objectives and providing informed consent.

The pilot sample consisted of five individuals who were not part of the final study population but shared similar educational and professional characteristics, namely trainees in family medicine and professionals in early childhood education. While they were not yet fully qualified professionals, they possessed sufficient academic and practical background to critically assess the relevance and clarity of the questionnaire items. Their feedback allowed for the identification and correction of potentially ambiguous, irrelevant, or culturally inappropriate items, ensuring that the final version would be more valid and accessible to the intended study participants. Importantly, the pilot participants' responses were not included in the study’s analytical dataset, as the purpose of the pilot was not to gather data for interpretation but to improve the instrument itself. Their insights led to minor wording adjustments and the clarification of certain items, but did not result in major structural changes. As such, while the pilot sample was not representative in a statistical sense, it played a methodological role in enhancing the internal validity of the questionnaire.

The final version of the instrument consisted of multiple-choice questions organized into three sections: demographic data to characterize participants; knowledge about ASD, assessing understanding of early signs, age of onset, diagnostic criteria, etiology, and prognosis; and professional experience with ASD, exploring frequency of contact with ASD cases and previous training. A section for open-ended comments, titled Observations/Suggestions, was also included to capture any additional reflections or clarifications that participants wished to share beyond the structured response format.

Descriptive statistical analysis was performed using IBM SPSS Statistics® software, version 21.0 for Windows (IBM Corp., Armonk, NY). Two-way frequency tables were used to examine associations between variables. The chi-square test was applied to determine whether significant differences existed between independent groups. A p-value of <0.05 was considered statistically significant.

The study was conducted in accordance with the ethical principles of the 2013 Declaration of Helsinki and adhered to all relevant ethical and legal standards. Approval was obtained from the local Ethics Committee of the Faculty of Health Sciences, University of Beira Interior, Covilhã and authorization to administer the questionnaire was granted by the Management Council of the Cova da Beira Health Center Group (ACeS) and by the directors of participating school groups (approval number: CE-FCS-2015-032). Questionnaires were completed in person between January and March 2016, following an explanation of the study’s purpose. Informed consent was obtained from all participants.

## Results

Four UCSPs agreed to participate, resulting in a response rate of 47.2% (n = 17) among 36 family physicians. Of the 12 school groupings contacted, seven agreed to participate, yielding responses from 21 kindergarten educators and 57 primary school teachers. The response rates for these groups were 56.8% (21/37) and 67.9% (57/84), respectively. The overall response rate across all participants was 60.5%.

A total of 95 professionals completed the questionnaire: 20% were male (n = 19) and 80% female (n = 76). The mean age was 51.5 years (range: 23-63 years). Regarding professional background, 57 were primary school teachers, 21 were kindergarten educators, and 17 were family physicians.

Table 1 presents the alarm signs identified by respondents, categorized by professional group. Most respondents were able to recognize common ASD warning signs. Statistically significant differences between professional groups were found for absence of imitation (χ²(4) = 16.06, p = 0.001, Cramér’s V = 0.29) and hypotonia/decreased muscle strength (χ²(2) = 9.2, p = 0.007, Cramér’s V = 0.22), both with small to moderate effect sizes. No significant associations were observed for other signs.

**Table 1:** "ASD is a chronic condition, affecting all domains of an individual's life, both short-term and long-term. Which do you consider to be part of the natural progression of the disorder?” (total n=95) NK: not known; NR: no response; Chi-2: chi-square; df: degrees of freedom; ASD: autism spectrum disorder

Signs of ASD	Family physicians (n=17)			Primary school teachers (n=57)			Kindergarten educators (n=21)						
	Yes	No	NK/NR	Yes	No	NK/NR	Yes	No	NK/NR	Chi-2	df	p-value	Cramér’s V
	n	n	n	n	n	n	n	n	n				
	%	%	%	%	%	%	%	%	%				
Lack of eye contact	14 (82.3%)	1 (5.9%)	2 (11.8%)	42 (73.7%)	8 (14.0%)	7 (12.3%)	19 (90.4%)	1 (4.8%)	1 (4.8%)	3.11	4	0.539	0.13
Hypotonia/decreased muscle strength	11 (64.7%)	5 (29.4%)	1 (5.9%)	12 (21%)	23 (40.4%)	22 (38.6%)	6 (28.6%)	6 (28.5%)	9 (42.9%)	9.2	2	0.007	0.22
Joint attention deficit	15 (88.2%)	0 (0%)	2 (11.8%)	54 (94.7%)	1 (1.8%)	2 (3.5%)	19 (90.5%)	0 (0.0%)	2 (9.5%)	2.59	4	0.535	0.12
Failure in reciprocal communication	15 (88.2%)	0 (0%)	2 (11.8%)	48 (84.2%)	5 (8.8%)	4 (7.0%)	19 (90.5%)	0 (0.0%)	2 (9.5%)	3.80	4	0.235	0.14
Frequent falls	1 (5.9%)	11 (64.7%)	5 (29.4%)	10 (17.5%)	23 (40.4%)	24 (42.1%)	3 (14.3%)	7 (33.3%)	11 (52.4%)	4.79	4	0.368	0.16
Excessive movement	8 (47.0%)	7 (41.2%)	2 (11.8%)	36 (63.2%)	13 (22.8%)	8 (14.0%)	12 (57.1%)	4 (19.1%)	5 (23.8%)	3.89	4	0.421	0.14
Delay/regression in language	12 (70.5%)	3 (17.7%)	2 (11.8%)	40 (70.2%)	9 (15.8%)	8 (14.0%)	13 (61.9%)	4 (19.1%)	4 (19.1%)	0.66	4	0.956	0.06
Impatience	9 (52.9%)	6 (35.3%)	2 (11.8%)	44 (77.2%)	8 (14.0%)	5 (8.8%)	16 (76.2%)	2 (9.5%)	3 (14.3%)	5.95	4	0.203	0.18
Absence of imitation	15 (88.2%)	2 (11.8%)	0 (0%)	23 (40.4%)	10 (17.5%)	24 (42.1%)	7 (33.3%)	6 (28.6%)	8 (38.1%)	16.06	4	0.001	0.29
Mood swings	10 (58.8%)	4 (23.5%)	3 (17.7%)	33 (57.9%)	7 (12.3%)	17 (29.8%)	14 (66.7%)	0 (0.0%)	7 (33.3%)	5.67	4	0.225	0.17
No response to voice/call	14 (82.4%)	1 (5.9%)	2 (11.8%)	39 (68.4%)	8 (14.0%)	10 (17.5%)	16 (76.2%)	1 (4.8%)	4 (19.1%)	2.41	4	0.660	0.11
Marked and persistent sadness	5 (29.4%)	7 (41.2%)	5 (29.4%)	19 (33.3%)	20 (35.1%)	18 (31.6%)	9 (42.9%)	3 (14.2%)	9 (42.9%)	3.97	4	0.410	0.14
Absence of emotional reciprocity	14 (82.3%)	2 (11.8%)	1 (5.9%)	36 (63.1%)	12 (21.1%)	9 (15.8%)	14 (66.7%)	3 (14.3%)	4 (19.1%)	2.76	4	0.694	0.12
Absence of symbolic play	13 (76.5%)	10 (5.9%)	3 (17.7%)	31 (54.4%)	8 (14.0%)	18 (31.6%)	9 (42.9%)	4 (19.0%)	8 (38.1%)	9.05	4	0.06	0.21
Aggressive behaviors	5 (29.4%)	7 (41.2%)	5 (29.4%)	34 (59.7%)	13 (22.8%)	10 (17.5%)	8 (38.1%)	5 (23.8%)	8 (38.1%)	7.78	4	0.096	0.2
Absence of pointing/showing gestures	9 (53.0%)	4 (23.5%)	4 (23.5%)	23 (40.4%)	14 (24.6%)	20 (35.1%)	8 (38.1%)	4 (19.1%)	9 (42.9%)	1.81	4	0.793	0.1
Preference for being alone	15 (88.2%)	0 (0%)	2 (11.8%)	45 (78.9%)	9 (15.8%)	3 (5.3%)	20 (95.2%)	0 (0.0%)	1 (4.8%)	7.5	4	0.112	0.2
Repetitive and purposeless movements	13 (76.4%)	2 (11.8%)	2 (11.8%)	50 (87.7%)	3 (5.3%)	4 (7.0%)	17 (81.0%)	2 (9.5%)	2 (9.5%)	1.54	4	0.819	0.09
Greater interest in inanimate objects	10 (58.8%)	1 (5.9%)	6 (35.3%)	21 (36.8%)	8 (14.0%)	28 (49.1%)	7 (33.3%)	4 (19.1%)	10 (47.6%)	3.63	4	0.458	0.14

In response to the question “At what age do the first symptoms of autism usually appear?”, the majority (n = 52) selected “before three years of age.” Among kindergarten educators, 28.6% (n=6) chose “between three and five years,” as did 38.6% (n=22) of primary school teachers and 41.2% (n=7) of family physicians. Only five of the total sample selected “between five and 10 years,” and none chose “after 10 years.” While all family physicians responded to the question, one kindergarten educator and two primary school teachers either did not respond or selected “I don't know.” No statistically significant association was found between profession and response choice (p = 0.718, χ²(2) = 2.0, Cramér's V = 0.10).

When asked, “Which of the following do you consider to be the main diagnostic criteria for autism spectrum disorder?”, the most frequently selected responses were “Persistent deficits in social communication and social interaction across multiple contexts” (n=80) and “Restricted and repetitive patterns of behavior, interests, or activities” (n=67). Other responses included “Presence of special abilities (e.g., numbers, memory)” (n=19), “Motor delay and coordination” (n=18), “Aggressive and destructive behavior” (n=17), “Lack of self-control” (n=14), and “Self-injury” (n=10). One person either did not respond or selected “I don’t know.” Notably, the combination of the two core diagnostic criteria was exclusively selected by 11 family physicians, 19 primary school teachers, and 11 kindergarten educators. No significant association was found between professional group and the selection of these combined criteria (p = 0.099, χ²(2) = 2.0, Cramér's V = 0.10).

In response to the question “What behavioral problems are specifically associated with autism?”, the most selected traits were “Resistance to change/alteration of routines” (n=85, 89.5%), “Poor eye contact” (n=74, 77.9%), and “Attention deficit” (n=61, 64.2%). Less frequently selected options included “Echolalia” (n=29, 30.5%), “Hyperactivity” (n=17, 17.9%), “Lack of common sense” (n=11, 11.6%), and “Criminal tendencies” (n=2). One respondent did not answer or selected “I don’t know.” When analyzing the exclusive selection of “Resistance to change,” “Echolalia,” and “Poor eye contact,” 77 participants did not make this association. By subgroup, 15 kindergarten educators, 48 primary school teachers, and 14 family physicians failed to associate these three key behaviors. No statistically significant difference was found between professions (p = 0.577, χ²(2) = 2.0, Cramér's V = 0.10).

In response to the question, “ASD is a multifactorial disorder. What factors do you think are involved?” 47 participants selected “Genetic and epigenetic factors,” 41 chose “Idiopathic,” and 34 indicated “Perinatal brain injury.” Approximately 11 of the respondents left the question unanswered. Less frequently selected factors included “Parenting problems” (n=7), “Postnatal brain injury” (n=6), and “Epilepsy” (n=2). Additionally, one participant selected “I don’t know,” and another noted “possible hereditary factors.” Regarding perceptions of ASD progression (Table 2), a statistically significant difference was observed for "Academic underachievement" (χ²(2) = 6.0, p = 0.026, Cramér’s V = 0.18), indicating a small-to-moderate effect. The belief that individuals with ASD require lifelong care and institutionalization was significantly more frequent among family physicians (χ²(4) = 38.21, p < 0.001, Cramér’s V = 0.45), reflecting a strong effect size. Other perceptions, such as the impact of early intervention on communication and autonomy, did not differ significantly between groups.

**Table 2:** "Regarding the suspicion of autism spectrum disorder (ASD), consider early warning signs" (total n=95) NK: not known; NR: no response; Chi-2: chi-square; df: degrees of freedom

Questions	Family physicians (n=17)			Primary school teachers (n=57)			Kindergarten educators (n=21)			Chi-2	df	p-value	Cramér’s V
	Yes	No	NK/NR	Yes	No	NK/NR	Yes	No	NK/ NR				
	n (%)	n (%)	n (%)	n (%)	n (%)	n (%)	n (%)	n (%)	n (%)				
Academic underachievement	14 (82.4%)	2 (11.7%)	1 (5.9%)	21 (36.8%)	22 (38.6%)	14 (24.6%)	11 (52.4%)	7 (33.3%)	3 (14.3%)	6.00	2	0.026	0.18
Need for curriculum adaptations and special education	16 (94.1%)	1 (5.9%)	0 (0.0%)	47 (82.5%)	4 (7.0%)	6 (10.5%)	20 (95.2%)	0 (0.0%)	1 (4.8%)	4.06	4	0.397	0.15
Delinquency	1 (5.9%)	12 (70.6%)	4 (23.5%)	2 (3.5%)	34 (59.7%)	21 (36.8%)	0 (0.0%)	12 (57.1%)	9 (42.9%)	2.43	4	0.657	0.11
Need for lifelong care and institutionalization	14 (82.4%)	2 (11.7%)	1 (5.9%)	7 (12.3%)	34 (59.7%)	16 (28.1%)	2 (9.5%)	13 (61.9%)	6 (28.6%)	38.21	4	0.363	0.45
Autonomous and independent life, but quite isolated	8 (47.1%)	5 (29.4%)	4 (23.5%)	16 (28.1%)	22 (38.6%)	19 (33.3%)	6 (28.6%)	7 (33.3%)	8 (38.1%)	2.56	4	0.634	0.12
Need for continuous support in employment and at home	10 (58.8%)	4 (23.5%)	3 (17.7%)	21 (36.8%)	12 (21.1%)	24 (42.1%)	9 (42.9%)	6 (28.6%)	6 (28.6%)	4.43	4	0.348	0.15
Need for psychiatric support, with frequent hospitalizations	1 (5.9%)	14(82.4%)	2 (11.7%)	3 (5.3%)	30 (52.6%)	24 (42.1%)	0 (0.0%)	12 (57.1%)	9 (42.9%)	6.66	4	0.155	0.19
Autonomous life without limitations, as they are more intelligent than the general population	2 (11.8%)	13 (76.5%)	2 (11.7%)	10 (17.5%)	23 (40.4%)	24 (42.1%)	3 (14.3%)	10 (47.6%)	8 (38.1%)	7.26	4	0.123	0.20
Early death	1 (5.9%)	9 (52.9%)	7 (41.2%)	3 (5.3%)	26 (45.6%)	28 (49.1%)	0 (0.0%)	9 (42.9%)	12 (57.1%)	1.86	4	0.762	0.10
Early intervention improves the prognosis of these patients	16 (94.1%)	0 (0.0%)	1 (5.9%)	47 (82.5%)	1 (1.8%)	9 (15.8%)	19 (90.5%)	1 (4.8%)	1 (4.8%)	3.55	4	0.471	0.14
Intervention improves autonomy, communication, and social interaction	16 (94.1%)	0 (0.0%)	1 (5.9%)	48 (84.2%)	1 (1.8%)	8 (14.0%)	20 (95.2%)	0 (0.0%)	1 (4.8%)	2.63	4	0.621	0.12
Intervention reduces stereotypies	11 (64.7%)	1 (5.9%)	5 (29.4%)	34 (59.7%)	1 (1.8%)	22 (38.6%)	12 (57.1%)	1 (4.8%)	8 (38.1%)	1.33	4	0.856	0.08

Additionally, most participants across all groups did not associate ASD with delinquency, early death, or full autonomy without limitations. Many selected “don’t know” or left those items unanswered. These results indicate a shared understanding of the importance of support and early intervention, but also reveal differing expectations regarding long-term outcomes based on professional role.

When asked whether they had ever worked with children diagnosed with ASD, 53.7% of respondents reported never having done so (n=51). This included six family physicians, 31 primary school teachers, and 14 kindergarten educators. “Some sporadic cases” was selected by six kindergarten educators, 25 primary school teachers, and 11 physicians. Only two of the total sample reported “many times,” none of whom were healthcare professionals.

A total of 87.4% of respondents reported having no specific training in ASD (n=83). Only one family physician indicated having training, mainly in neurodevelopment, as did one kindergarten educator and one primary school teacher. Additionally, two kindergarten educators and seven primary school teachers reported having training in special education. 

As shown in Table 3, no family physician strongly agreed with feeling capable of identifying early warning signs of ASD, despite many acknowledging their role as key professionals in early detection. In the open-ended “Observations” section, participants across all professions expressed interest in receiving training in this area.

**Table 3:** Personal and professional experience with ASD (total n=95) Chi-2: chi-square; df: degrees of freedom; ASD: autism spectrum disorder

Questions	Groups		Strongly disagree	Somewhat disagree	Neither agree nor disagree	Somewhat agree	Strongly agree	Not applicable/Did not respond	Chi-2	df	p-value	Cramér’s V
I feel confident in identifying signs of alarm for ASD	Family physicians (n=17)	n (%)	9 (52.9%)	2 (11.8%)	5 (29.4%)	1 (5.9%)	0 (0%)	0 (0%)	13.00	10	0.224	0.26
	Primary school teachers (n=57)	n (%)	17 (29.8%)	8 (14%)	20 (35.1%)	5 (8.8%)	5 (8.8%)	2 (3.5%)				
	Kindergarten educators (n=21)	n (%)	8 (38.1%)	0 (0%)	8 (38.1%)	0 (0%)	2 (9.5%)	3 (14.3%)				
I feel comfortable administering ASD screening tests (M-CHAT, CARS...)	Family physicians (n=17)	n (%)	11 (64.7%)	3 (17.7%)	2 (11.8%)	0 (0%)	0 (0%)	1 (5.9%)	5.47	10	0.857	0.17
	Primary school teachers (n=57)	n (%)	30 (52.6%)	4 (7%)	10 (17.5%)	1 (1.8%)	2 (3.5%)	10 (17.5%)				
	Kindergarten educators (n=21)	n (%)	12 (57.1%)	3 (14.3%)	3 (14.3%)	0 (0%)	1 (4.8%)	2 (9.5%)				
I believe all children should undergo formal screening	Family physicians (n=17)	n (%)	8 (47.1%)	3 (17.7%)	2 (11.8%)	0 (0%)	4 (23.5%)	0 (0%)	7.69	10	0.659	0.20
	Primary school teachers (n=57)	n (%)	22 (38.6%)	6 (10.5%)	12 (21.1%)	3 (5.3%)	9 (15.8%)	5 (8.8%)				
	Kindergarten educators (n=21)	n (%)	6 (28.6%)	2 (9.5%)	5 (23.8%)	3 (14.3%)	3 (14.3%)	2 (9.5%)				
I feel that I am in a privileged position to early identify these cases	Family physicians (n=17)	n (%)	1 (5.9%)	1 (5.9%)	4 (23.5%)	2 (11.8%)	9 (52.9%)	0 (0%)	13.64	10	0.19	0.27
	Primary school teachers (n=57)	n (%)	7 (12.3%)	2 (3.5%)	19 (33.3%)	10 (17.5%)	14 (24.6%)	5 (8.8%)				
	Kindergarten educators (n=21)	n (%)	1 (4.8%)	0 (0%)	3 (14.3%)	2 (9.5%)	13 (61.9%)	2 (9.5%)				
I feel comfortable providing support to these children	Family physicians (n=17)	n (%)	10 (58.8%)	4 (23.5%)	1 (5.9%)	1 (5.9%)	0 (0%)	1 (5.9%)	7.77	10	0.651	0.20
	Primary school teachers (n=57)	n (%)	25 (43.9%)	10 (17.5%)	9 (15.8%)	7 (12.3%)	1 (1.8%)	5 (8.8%)				
	Kindergarten educators (n=21)	n (%)	8 (38.1%)	2 (9.5%)	7 (33.3%)	2 (9.5%)	0 (0%)	2 (9.5%)				
I believe I would benefit from specific training in this area	Family physicians (n=17)	n (%)	0 (0%)	0 (0%)	0 (0%)	0 (0%)	15 (88.2%)	2 (11.8%)	7.88	10	0.641	0.20
	Primary school teachers (n=57)	n (%)	2 (3.5%)	3 (5.3%)	4 (7.0%)	3 (5.3%)	40 (70.2%)	5 (8.8%)				
	Kindergarten educators (n=21)	n (%)	1 (4.8%)	0 (0%)	2 (9.5%)	1 (4.8%)	17 (81.0%)	0 (0%)				

## Discussion

This study aimed to assess the general knowledge and perceived challenges of family physicians, primary school teachers, and kindergarten educators in Portugal in identifying ASD.

Participants generally demonstrated an ability to recognize the core early warning signs of ASD. However, there was considerable uncertainty regarding less obvious signs such as the absence of proto-declarative gestures, symbolic play, and imitation, recognized indicators of the disorder. Additionally, a high proportion of respondents misidentified symptoms not typically associated with ASD, such as frequent falls, impatience, mood swings, and persistent sadness, suggesting gaps in theoretical knowledge.

A 2015 study by Al-Sharbati et al. [25], involving 164 primary school teachers in Oman, found that 55% of respondents recognized poor eye contact as a sign of ASD, compared to 73% among the primary school teachers in our sample. Although useful for contextualization, cross-cultural comparisons must be interpreted with caution due to differences in professional training and educational systems.

Given the early onset of ASD symptoms, family physicians and kindergarten educators are in a privileged position to contribute to timely identification, enabling appropriate referral, social inclusion, and educational support. However, approximately 45% of respondents did not recognize the possibility of diagnosis before the age of 3, indicating a limited awareness of the early manifestations of the disorder. When evaluating participants’ responses, it is important to consider what constitutes the “gold standard” for ASD knowledge. In this context, pediatricians with specific training in neurodevelopmental disorders may be a more appropriate benchmark than general practitioners or educators.

Most participants correctly identified at least one of the core diagnostic criteria of ASD, particularly deficits in communication and restricted, repetitive behavior. The majority also acknowledged that early intervention improves prognosis, autonomy, communication, and social interaction. However, when asked about their confidence in identifying early warning signs, most physicians strongly disagreed. These findings highlight the need for continuous training among primary care providers regarding risk factors, screening tools, and evidence-based management strategies for ASD.

Interestingly, no family physician in the sample strongly agreed with feeling capable of identifying early warning signs, despite acknowledging their role in early detection. This may be partially explained by limited clinical exposure to diagnosed cases. However, it is important to consider that this does not necessarily reflect a genuinely low prevalence of ASD in the country. Global estimates place ASD prevalence at approximately one in 100 children, a figure that likely applies to Portugal as well [26]. Therefore, it is plausible that what is low is not the actual prevalence but rather the rate of diagnosis, potentially due to insufficient training, under-recognition of early signs in primary care, or delays in referral to specialists. While some argue that improved identification may inflate prevalence estimates, the available data suggests that increased access to diagnostic services and broader educational support, rather than an actual rise in incidence, better explain the observed trends in ASD diagnosis.

School integration is a fundamental component of early intervention in ASD, as it fosters both learning and socialization. Training for educators, both in special and general education, and promoting awareness within the school community are essential to creating inclusive environments and improving outcomes for children with ASD.

Participants who felt confident in identifying early signs were also more likely to report confidence in providing support. Those who felt capable of recognizing early symptoms were also more likely to feel competent in providing appropriate follow-up and care, reflecting a degree of self-awareness regarding their professional limitations.

Most participants expressed interest in receiving ASD-specific training. A pilot study conducted in São Paulo, Brazil [27], evaluated a training program consisting of three hours per week, two hours of lectures and one hour of case discussions with ASD specialists, designed to enhance early detection skills among primary care providers. Pre- and post-intervention assessments demonstrated promising improvements in participants’ knowledge and diagnostic confidence. Future training initiatives should be tailored to the specific context of each professional group. For example, short online modules or case-based workshops may be particularly effective for family physicians, while school-based professional development sessions may be more appropriate for educators. Interdisciplinary training formats, involving both healthcare and education professionals, could also promote a shared understanding of early detection practices and facilitate better communication between sectors.

One of the main limitations of this study is its small sample size and restricted geographical scope, as all participants were from the same region of Portugal. This limits the generalizability of the findings to other regions or contexts. Although the sample was heterogeneous in terms of professional background, the uneven distribution across groups and the lack of broader geographic and institutional representation are acknowledged limitations. Future studies should aim to expand both the size and diversity of the sample, incorporating participants from multiple regions and, where possible, other countries, and should consider reassessing knowledge following structured training interventions.

What is known?

Early identification of ASD is crucial for improving prognosis, autonomy, and social functioning. Family physicians, primary school teachers, and kindergarten educators are key professionals in the early recognition of ASD symptoms. A persistent knowledge gap regarding ASD diagnostic criteria and early warning signs has been documented across these groups.

What is added?

This study highlights that many professionals in healthcare and education lack confidence in recognizing early signs of ASD. It identifies limited awareness of the typical age of symptom onset, with most respondents expecting diagnosis only after age three. A significant unmet need for ASD-specific training was reported, supporting the urgency of tailored education across both sectors.

## Conclusions

No significant differences were found between professional groups regarding overall ASD knowledge, highlighting a widespread need for training. While most participants successfully identified the main warning signs of ASD, recognition of less typical or early signs, such as the absence of proto-declarative gestures, symbolic play, or imitation, was notably lower. Nearly half of the respondents believed that diagnosis typically occurs only after the age of three, despite clinical evidence indicating that symptoms are often observable between 18 and 24 months. These findings point to areas where additional training may be beneficial. However, it is important to interpret this cautiously and avoid placing undue responsibility on professionals without considering the broader systemic context. The data revealed consistent knowledge gaps across all professional groups, particularly in the areas of early detection, practical experience, and formal training. Most respondents reported limited contact with children diagnosed with ASD, low confidence in identifying early signs, and a lack of specific training. Nonetheless, there was a clear and widespread interest in receiving further education on the topic.

It is important to consider that limited professional exposure to ASD may reflect not only individual knowledge gaps but also broader structural or regional factors, such as lower local diagnosis rates, reduced parental demand for formal evaluation, or the existence of alternative diagnostic pathways that bypass primary care or educational settings. In this context, suggesting that "more training" is the sole solution would be reductive. Professional engagement with ASD is shaped by multiple interdependent factors, including visibility of the condition, institutional priorities, policy frameworks, and the degree of family advocacy. Without clear signals that ASD is a prevalent and urgent concern in their community, it is understandable that professionals may prioritize other pressing demands within their scope of work. Future studies should explore these dynamics in greater depth, particularly whether low reported contact reflects genuinely low incidence, delayed or missed diagnoses, or under-recognition. It would also be important to examine whether current diagnostic pathways limit the involvement of frontline professionals and how local contexts, both social and institutional, shape training needs and clinical awareness. Expanding this research on a national scale would help assess ASD awareness more comprehensively, not only among healthcare and education professionals, but also within the general population. Improving societal awareness, along with coordinated educational and medical support, remains essential for fostering early identification, timely intervention, and inclusion.

## Figures and Tables

**Figure 1:**
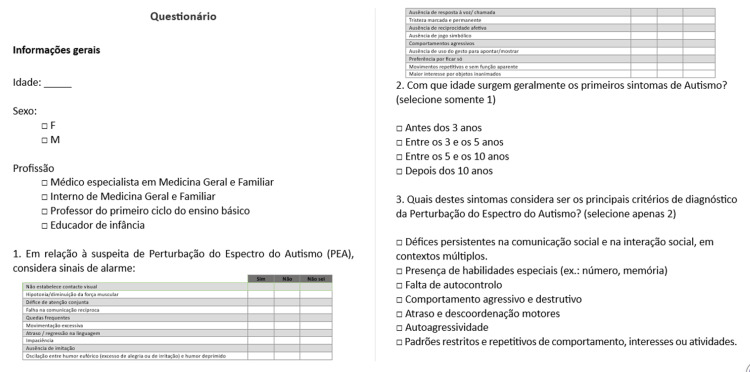
Questionnaire: part 1

**Figure 2:**
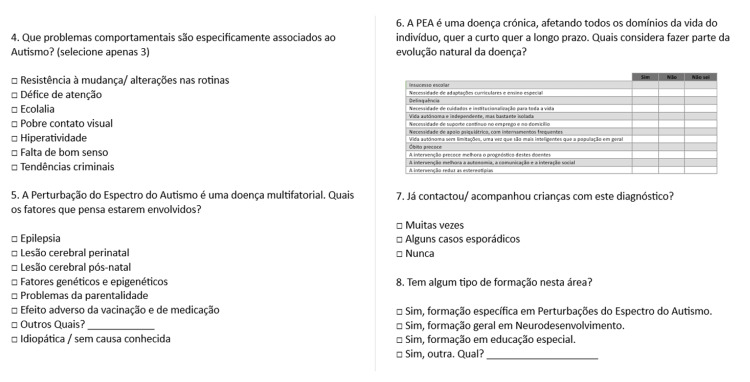
Questionnaire: part 2

**Figure 3:**
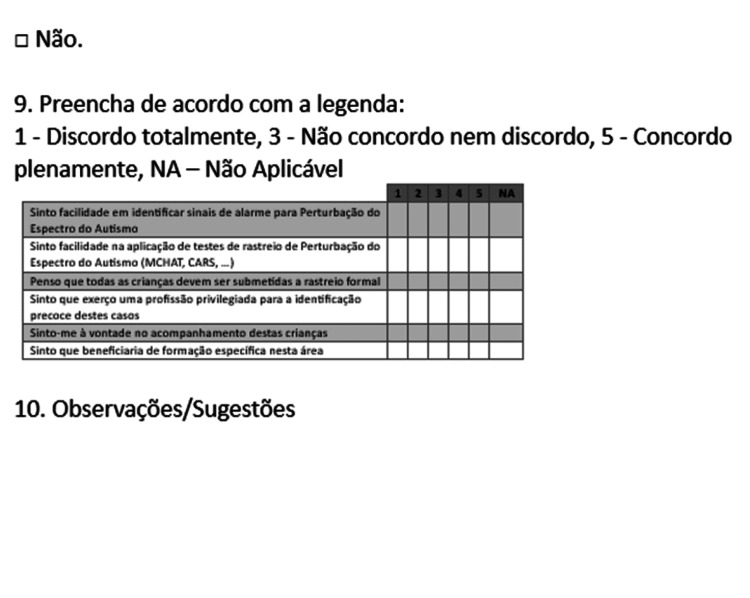
Questionnaire: part 3

**Figure 4:**
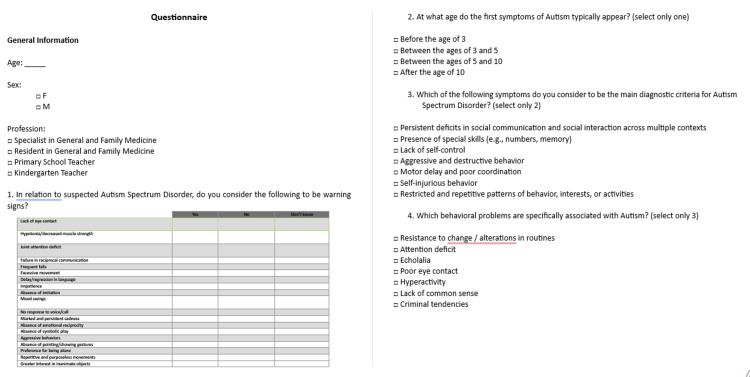
English questionnaire: part 1

**Figure 5:**
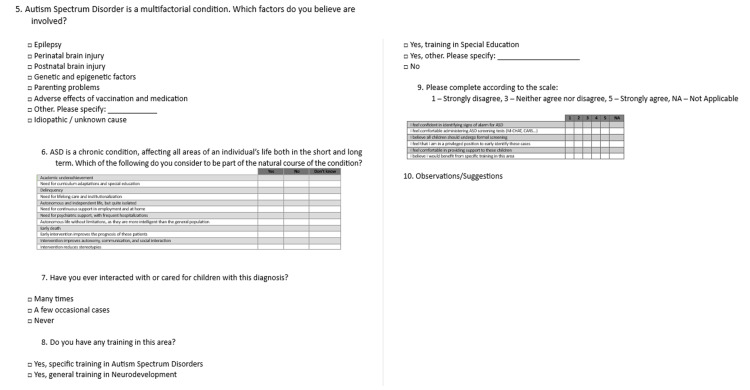
English questionnaire: part 2

## Appendices

Appendix A

Questionnaire in Portuguese

Appendix B

Questionnaire in English
